# Ebola and Its Control in Liberia, 2014–2015

**DOI:** 10.3201/eid2202.151456

**Published:** 2016-02

**Authors:** Tolbert G. Nyenswah, Francis Kateh, Luke Bawo, Moses Massaquoi, Miatta Gbanyan, Mosoka Fallah, Thomas K. Nagbe, Kollie K. Karsor, C. Sanford Wesseh, Sonpon Sieh, Alex Gasasira, Peter Graaff, Lisa Hensley, Hans Rosling, Terrence Lo, Satish K. Pillai, Neil Gupta, Joel M. Montgomery, Ray L. Ransom, Desmond Williams, A. Scott Laney, Kim A. Lindblade, Laurence Slutsker, Jana L. Telfer, Athalia Christie, Frank Mahoney, Kevin M. De Cock

**Affiliations:** Ministry of Health and Social Welfare, Monrovia, Liberia (T.G. Nyenswah, F. Kateh, L. Bawo, M. Massaquoi, M. Gbanyan, M. Fallah, T.K. Nagbe, K.K. Karsor, C.S. Wesseh, S. Sieh, H. Rosling);; World Health Organization, Monrovia (A. Gasasira);; United Nations Mission for Emergency Ebola Response, Monrovia (P. Graaff);; National Institutes of Health, Bethesda, Maryland, USA (L. Hensley);; Karolinska Institute, Stockholm, Sweden (H. Rosling);; Centers for Disease Control and Prevention, Atlanta, Georgia, USA (T. Lo, S.K. Pillai, N. Gupta, J.M. Montgomery, R.L. Ransom, D. Williams, A.S. Laney, K.A. Lindblade, L. Slutsker, J.L. Telfer, A. Christie, F. Mahoney, K.M. De Cock)

**Keywords:** Ebola, Liberia, response, hemorrhagic fever, Ebola virus disease, epidemic, outbreak, public health, health systems, viruses

## Abstract

The severe epidemic of Ebola virus disease in Liberia started in March 2014. On May 9, 2015, the World Health Organization declared Liberia free of Ebola, 42 days after safe burial of the last known case-patient. However, another 6 cases occurred during June–July; on September 3, 2015, the country was again declared free of Ebola. Liberia had by then reported 10,672 cases of Ebola and 4,808 deaths, 37.0% and 42.6%, respectively, of the 28,103 cases and 11,290 deaths reported from the 3 countries that were heavily affected at that time. Essential components of the response included government leadership and sense of urgency, coordinated international assistance, sound technical work, flexibility guided by epidemiologic data, transparency and effective communication, and efforts by communities themselves. Priorities after the epidemic include surveillance in case of resurgence, restoration of health services, infection control in healthcare settings, and strengthening of basic public health systems.

## Abstract 2

Several factors explain the successful response to the outbreak in this country.

In Liberia, Ebola virus disease was first reported from Lofa County on March 30, 2014, a week after cases in Guinea had been reported (*1*–*3*) (Figure 1). Additional cases in May and June heralded the country’s severe outbreak (*4*). Events in Liberia drew widespread attention to Ebola as a threat to global health security (*5*) including urbanization of the disease; first-ever infections in expatriate health workers (*6*); international spread to Nigeria, the United States, and Spain with secondary transmission (*7*–*9*); and mathematical model estimates of a future high case load (*10*).

**Figure 1:**
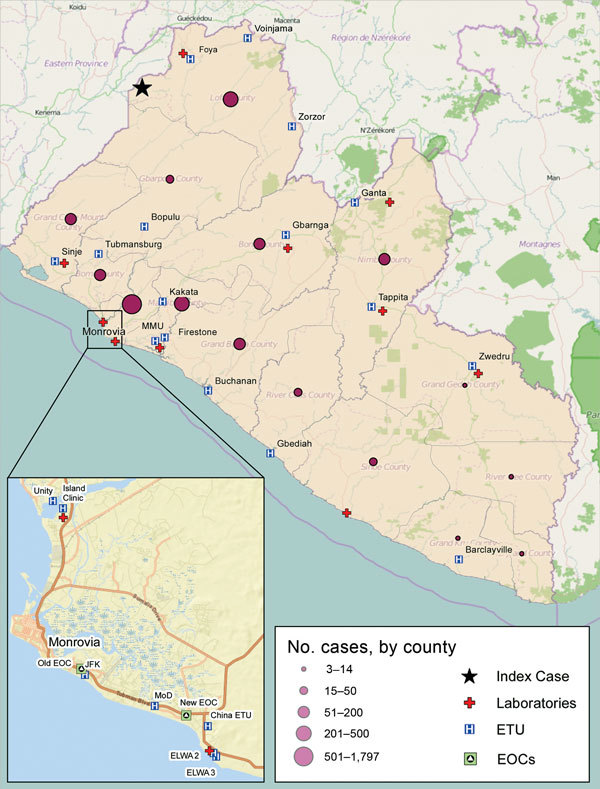
Locations of Ebola case-patients and associated facilities, Liberia, 2014–2015. ELWA, Eternal Love Winning Africa; EOC, emergency operations center; ETU, Ebola treatment unit; JFK, John Fitzgerald Kennedy; MoD, Ministry of Defense.

On August 4, 2014, the US ambassador to Liberia declared a disaster; on August 6, the president of Liberia declared a state of emergency; and on August 8, the World Health Organization (WHO) called Ebola in West Africa a public health emergency of international concern (*11*). Ten months later, on May 9, 2015, WHO declared Liberia free of Ebola virus transmission (*12*). However, on June 29, 2015, a postmortem diagnosis of Ebola was made for a 17-year-old boy, and 5 other cases were subsequently confirmed, but no further spread was noted. Liberia was again declared free of Ebola on September 3, 2015 (*13*). We describe the Ebola experience in Liberia and draw conclusions relevant to future responsiveness.

## Incident Management System and Coordination of the International Response, July–September, 2014

**Figure 2:**
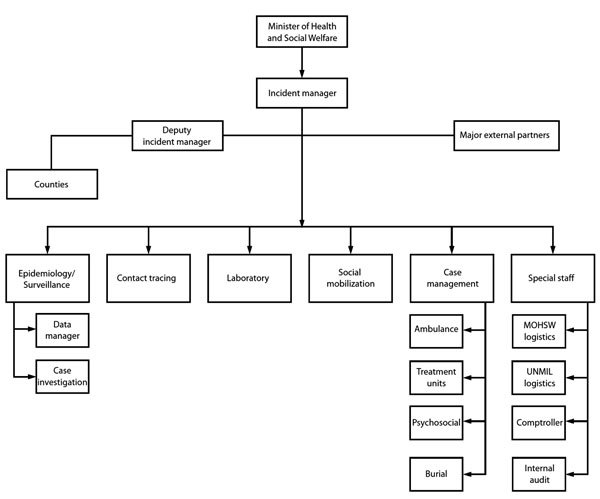
Organizational flowchart for Ebola response Incident Management System, Liberia Ministry of Health and Social Welfare (MOHSW), August 2014. UNMIL, United Nations Mission in Liberia. Source: http://www.cdc.gov/mmwr/preview/mmwrhtml/mm6341a4.htm

The government of Liberia initially set up a diverse Ebola Task Force, whose large size and organizational challenges handicapped its effectiveness. In late July 2014, supported by the US Centers for Disease Control and Prevention (CDC), WHO, and other partners, the Liberia Ministry of Health and Social Welfare (MOHSW) implemented an Incident Management System (IMS) with an incident manager devoted exclusively to Ebola (*14*). The IMS ensured streamlined management, clear authority and accountability, structured working groups, and operational follow-up. In September 2014, the IMS moved into an emergency operations center, a location for coordination and oversight of all operations. The incident manager had a deputy empowered to deal with logistics and operational issues and, eventually, an inner core of advisors (including persons from WHO, CDC, the US Agency for International Development, and the UN Mission for Ebola Emergency Response) who conferred daily to coordinate activities. International partners co-chaired IMS technical work groups: case management, contact tracing, safe burials, surveillance, laboratory, and social mobilization (Figure 2). The president of Liberia interacted directly with the incident manager. Separately, the president convened the Presidential Advisory Committee on Ebola, a small group of senior officials and international partners who provided advice about sensitive matters and policy.

## Surveillance, Epidemiology, and Laboratory Diagnosis, July 2014–May 2015

An early priority for directing the response was surveillance. Using WHO case definitions for suspected, probable, and confirmed cases, the 15 counties of Liberia reported Ebola cases to MOHSW. Reporting modalities included case investigation forms; mobile phone, text, and email messages; reports from Ebola Treatment Units (ETUs); laboratory results; and reports from burial teams, case investigators, and contact tracers. Timely reconciliation of data from multiple sources proved challenging. Constraints included shortage of trained staff, lack of communication and information technology, poor internet and mobile phone coverage, and lack of transport from remote locations.

Choice of data management platforms proved difficult. Initially, case data were entered into an application based on Epi Info version 7, developed by CDC for hemorrhagic fever outbreaks (https://epiinfovhf.codeplex.com). However, in the face of a widespread epidemic, this software had limitations. In mid-December 2014, MOHSW changed to District Health Information Software 2 (https://www.dhis2.org/), an open-source software platform enabling web-based data entry. Contact tracing generally depended on paper and Excel (Microsoft, Redmond, WA, USA) spreadsheets maintained at the county level. At the national level, daily situation reports were compiled manually from aggregate data received from the counties. Although these reports were instrumental in guiding the response, they were incomplete, contained duplicates, and could not be analyzed in real time. Reconciliation of all available data is ongoing.

In July 2014, only 1 laboratory, at the Liberia Institute for Biomedical Research outside Monrovia, was able to conduct Ebola testing, with support from the US National Institutes of Health and the US Army Medical Research Institute of Infectious Diseases (*5*). International partners, including the US military, established a temporary laboratory network to provide Ebola test results within 24 hours to anywhere in the country. By December 2014, real-time reverse transcription PCR testing for Ebola genomic RNA was available at 10 laboratories nationwide. Throughout the outbreak, adequate staffing and rapid transport for specimens, such as by helicopter from remote areas, remained challenging.

## Patient Isolation, Case Management, and Epidemic Trends, July–November 2014

The IMS emphasized 4 pillars for interrupting Ebola transmission: 1) early detection, isolation, and treatment of cases; 2) safe transport of patients with suspected cases; 3) safe burial; and 4) infection prevention and control (IPC) in healthcare settings. Isolating persons with Ebola was an immediate, overriding objective. Initially, contact tracing was difficult because of the large number of cases and the urgent need to isolate patients and dispose of cadavers.

By mid-July 2014, only 2 ETUs (20 beds each) were operational, in Foya (Lofa County) and Monrovia (Montserrado County). The principal organizations working with MOHSW to provide care for Ebola patients were Médecins Sans Frontières (MSF) and Samaritan’s Purse, but Samaritan’s Purse withdrew after several of their staff members became infected with Ebola virus (*6*). By the end of July 2014, the country faced a crisis as the ETUs were filled beyond capacity and Ebola patients were turned away, often dying on hospital grounds, in city streets, or in their homes. In mid-August 2014, MSF opened a tented 120-bed ETU in Monrovia, the largest ever built, with capacity to expand to 400 beds (*15*). The rapid increase in Ebola cases (*5*), extension of the epidemic to all counties, and projections from mathematical modeling (*10*) led the IMS to envision ETUs nationwide. Although a total of 27 ETUs with >2,000 beds were planned, ultimately, 25 were built, of which 3 never opened and many remained underutilized as the epidemic waned.

In early August 2014, intense discussions occurred with MOHSW about the level of care that limited staff could safely provide. Partners including MSF, CDC, and Samaritan’s Purse concluded that care should be simplified and that often only oral instead of intravenous rehydration could be safely provided (MSF and CDC, unpub. data; [*5*]). Partners also considered management in the community if patients were unwilling or unable to be evacuated. Lower level community care centers (CCCs) were developed to meet the urgent need for local isolation facilities before sufficient ETUs were constructed (*15*,*16*). However, capacity to build CCCs was also limited and impeded by concern for inferior care or safety. Although >80 CCCs were envisaged, <10 became operational.

**Figure 3:**
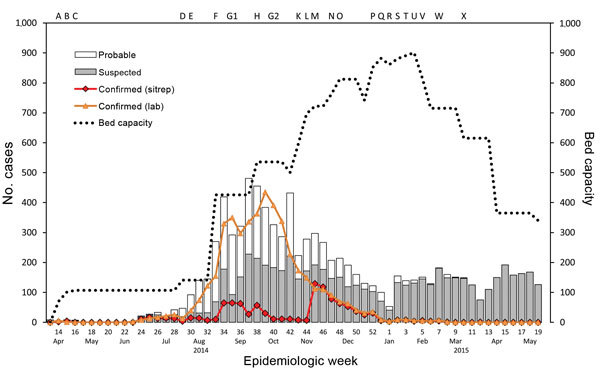
Trends over time for suspected, probable, and confirmed cases of Ebola virus disease from situation reports (sitreps); for confirmed cases from laboratory reports (lab); and for numbers of Ebola treatment unit beds, Liberia 2014–2015. Ebola treatment unit build completion: A, Foya; B, Firestone; C, Eternal Love Winning Africa (ELWA) 1; D, ELWA2; F, ELWA3, John Fitzgerald Kennedy Hospital; H, Bong, Island; K, Unity; L, Ministry of Defense; M, Monrovia Medical Unit; N, Bomi, Kakata; O, China; P, Buchanan; SKD*; Q, Sinje, Ganta, Gbediah; R, Bopulu; S, Tappita, Zwedru; T, Voinjama; U, Zorzor, Greenville*; V, Barclayville; W, Fishtown*; X, Harper.* Other response events: E, Incident Management System implemented; G1–G2, burial teams trained and deployed. *Ebola treatment units built but never opened.

**Figure 4:**
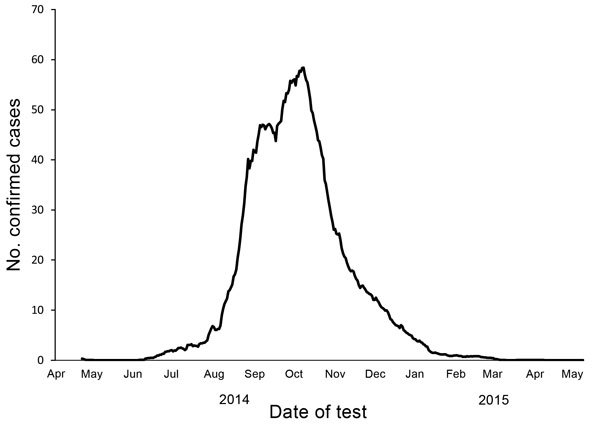
Epidemic curve for laboratory-confirmed cases of Ebola virus disease, Liberia, April 2014–May 2015. Confirmed cases were based on laboratory data per 21-day moving average.

The number of available ETU beds initially lagged behind need, but by late September 2014, bed capacities exceeded new cases (Figure 3) (*17*). In retrospect, different data sources suggest that the incidence of disease that had started to increase exponentially in June peaked in early October 2014 and that during July–August, the epicenter shifted from Lofa County to Montserrado County (*5**,**18*). By November 2014, the epidemic was characterized by low numbers of cases overall, about half in Monrovia and the rest in small clusters in remote locations across the country, frequently initiated by infected travelers from the capital (*19**,**20*). Lower case counts and increased staff facilitated data reconciliation. Manual matching of laboratory results with ETU and burial data became logistically feasible. Although incomplete, verified laboratory data proved the most useful indicator of epidemic trends (Figure 4). Data from ETUs, although not capturing all cases, provided descriptive characteristics of persons with Ebola.

## Management of Cadavers, July–December 2014

The International Federation of Red Cross and Red Crescent Societies and the nongovernmental organization Global Communities led safe collection and disposal of cadavers, a culturally sensitive issue. Starting in September 2014, initial efforts in Lofa and Montserrado Counties were expanded nationwide. Ebola testing of postmortem blood or oral swab samples enabled detection of unrecognized Ebola cases and assessment of excess deaths resulting from Ebola (*21*). In Monrovia, swampy topography and heavy rains in early August 2014 led to resurfacing of recently buried bodies, causing public outrage. The president of Liberia decreed mandatory cremation, a taboo that was accepted reluctantly and incompletely. The decree was lifted in late December 2014 when a public cemetery was opened outside the capital.

**Figure 5:**
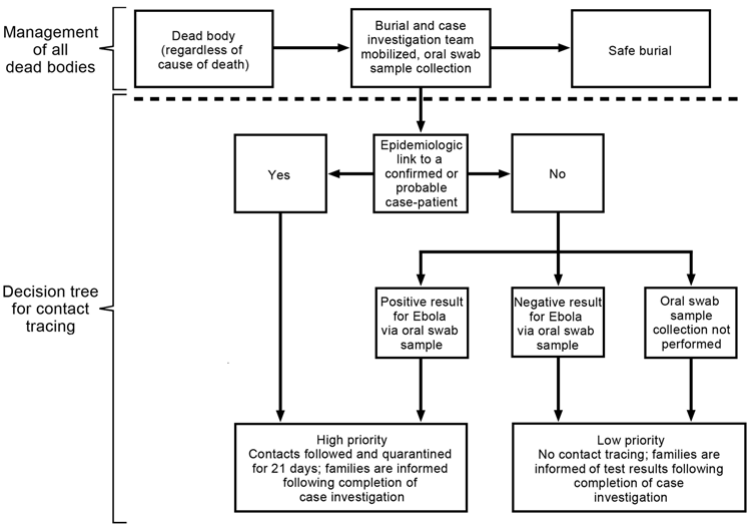
Proposed algorithm for management of dead bodies and associated contact tracing for Ebola virus disease, Liberia, 2014–2015.

Ebola virus–positive cadavers in Montserrado County peaked at 380 during the week of September 15, 2014 (*22*). From October to December, the estimated proportion of Ebola virus–positive cadavers in Montserrado County declined from 35% to 5%. However, the proportion of all bodies collected by burial teams was estimated at <50%, even lower outside of Montserrado County. An algorithm to use cadaver swab sample results to guide contact tracing was extensively discussed but incompletely adopted (Figure 5).

## Ebola in Healthcare Workers and IPC, July 2014–May 2015

**Figure 6:**
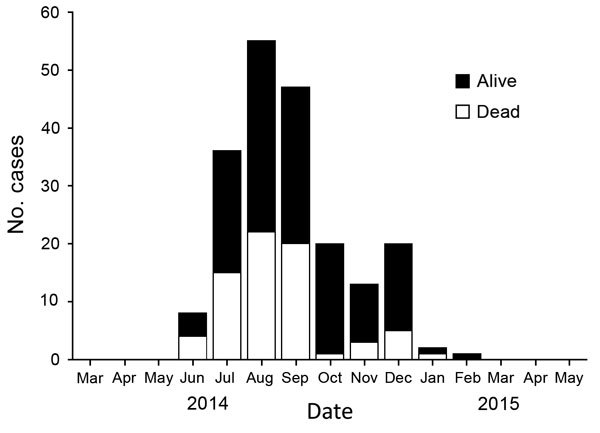
Trends for reported Ebola virus infections among 202 healthcare workers, by status and month, Liberia, March 2014–May 2015. Data source: daily aggregate reports of new cases in healthcare workers in Liberia and Liberia Ministry of Health and Social Welfare situation reports.

Early investigations demonstrated greatly increased risk for Ebola among healthcare workers (*5*,*23*), who accounted for 97 (12%) of 810 cases reported by mid-August 2014 (*23*). The greatest proportions of cases were in nurses and nurse aides (34/97; 35%) and physicians and physician assistants (17/97; 18%) (*23*). Most healthcare worker infections were acquired outside ETUs. During July–August 2014, a total of 11 clusters of Ebola involving healthcare workers were investigated; only 1 was in persons working in an ETU (*23*), but even for those in that cluster, exposure in the adjacent hospital was considered probable (*6*). Early estimates were that only ≈25% of Ebola patients received treatment in ETUs (*23*), and the overall proportion of healthcare worker infections thought to have been acquired in ETUs was only 2.4%; the rest were acquired in general hospitals or clinics, including informal venues (MOHSW, unpub. data through December 9, 2014) Trends in reported Ebola infections in healthcare workers are shown in Figure 6). Over the course of the epidemic in Liberia, 378 healthcare workers had confirmed cases of Ebola and 192 died (case-fatality rate 50.8%). These numbers represent 12% (378/3,157) of all confirmed cases and 4% (192/4,808) of all Ebola deaths.

Weak IPC rendered all 657 healthcare facilities in Liberia vulnerable (*5*). Most facilities where Ebola transmission occurred subsequently closed down. A national IPC task force was established, and an IPC strategy was developed to guide MOHSW, assess facilities and their needs, provide standardized training (Keep Safe, Keep Serving), and conduct investigations. Of the 79 healthcare facilities surveyed before the end of 2014, an estimated 57% lacked protocols for triage and isolation of persons suspected to have Ebola; 43% did not have access to gloves, face shields, or gowns; and 24% lacked running water (MOHSW, unpub. data). IPC committees in healthcare facilities were universally lacking.

By the end of 2014, >4,000 healthcare workers from 350 facilities had received training in basic IPC. A cadre of physicians were trained to serve as technical advisors in the counties. IPC focal points for major hospitals were selected and trained; surveillance and investigative capacity for Ebola in healthcare workers was developed; and personal protective equipment was delivered to major facilities nationwide (gloves and bleach were made as widely available as possible).

The value of surveillance among healthcare staff was highlighted by a single transmission chain in early 2015, in which 166 non-ETU healthcare workers at 10 facilities were exposed to the virus; remarkably, only 1 healthcare worker became infected (*24*). An innovative intervention in response to this cluster was the ring IPC strategy, which provided intensified IPC training and support to healthcare facilities around areas of active transmission (*25*). Despite impressive accomplishments during the epidemic, enormous challenges and deficiencies remain.

## Social Mobilization and Public Communication, August 2014–March 2015

MOHSW led comprehensive social mobilization to educate the public on the signs and symptoms of Ebola and provide essential health protection information. Because Ebola was new to Liberia, the first communication strategy comprised messages to counter disbelief (e.g., Ebola is Real). As fear of Ebola and stigma increased, hiding illness became common, prompting messages to encourage help-seeking behavior.

Liberia has a strong tradition of oral communication; therefore, thousands of general community health volunteers were trained to share health messages locally. In October 2014, traditional leaders convened and resolved to support government interventions, opening another trusted channel of health information. During November 2014, traditional and community leaders supported training in all of Liberia’s 88 districts. Novel methods were instituted, such as providing traditional chiefs with mobile phones to report suspected cases.

By December 2014, when cases were fewer and response capacity was more robust, a national campaign to reduce Ebola incidence to zero was declared. The evidence-based “Ebola Must Go!” campaign defined 5 essentials in commonly used language: safe burial, rapid isolation of suspected cases, provision of treatment, identification and 21-day monitoring of contacts, and encouragement to speak out against concealment of illness.

## Getting to Zero in a Declining Epidemic, Mid-November 2014–May 2015

Starting in mid-November 2014, several events heralded the waning of the epidemic in Liberia. About half of Ebola cases were now part of discrete rural outbreaks often affecting villages so remote that even motorcycles could not reach them. Helicopter airlift was limited by restricted US military air deployments and delays in commercial contracts. Epidemiologic field teams faced harsh living conditions and challenging logistics, often having to walk long distances to and from settlements.

The IMS promoted the Rapid Isolation and Treatment of Ebola (RITE) strategy, which empowered county authorities, with support from partners, to respond quickly to remote hot spots (*22**,**23*). Essentials were engagement of community leaders, community education, active case finding and contact tracing, quarantine of high-risk contacts, isolation and care for patients, and safe burials. Voluntary quarantine and ad hoc rudimentary clinical facilities were used as needed; emphasis was placed on moving suspected case-patients and contacts from remote locations closer to ETUs. In Bong County, tents were erected in a football stadium to shelter and feed >40 contacts during their 21-day monitoring period.

Of 15 remote outbreaks in 2014, detailed analyses were conducted for 12 (in 9 counties) (*20*), of which 9 gave insight into transmission dynamics (*26*). After implementation of the RITE strategy, clusters were recognized earlier, greater proportions of patients were isolated and diagnoses laboratory confirmed, transmission chains became fewer, durations of outbreaks shortened, and case-fatality proportions declined (*20**,**26*). The 0–27 secondary cases before RITE declined to 0–4, and the number of secondary cases generated by 1 primary case (basic reproduction number) decreased by 94% (*26*).

Application of fundamental principles—suspect case isolation, rapid diagnosis, and contact tracing—yielded results but required different tactical approaches in varied settings. Ebola had become less widespread, enabling recognition of individual transmission chains in a way not possible earlier. By December 2014, <10 cases were being reported daily, and focus turned to case investigation and contact tracing around laboratory-confirmed cases. In December 2014, Montserrado County set up its own IMS. A sector approach was introduced to decentralize activities in Monrovia, dividing the city into 4 sectors in which partners supported an integrated response for smaller populations.

Considerable resources were invested in containing the last known transmission chains. The Saint Paul’s Bridge cluster (22 cases in Monrovia, 15 fatal) was characterized by 3 generations of transmission and challenging social circumstances including resistance, poverty, urban gangs, substance abuse, and extensive exposures in healthcare settings. The last patient in the cluster was isolated on February 18, 2015, and discharged with negative Ebola test results in early March.

Liberia was ready to be declared free of Ebola when on March 20, 2015, an Ebola diagnosis was confirmed for a 44-year-old woman in Monrovia, who died 1 week later (*27*). Her most likely exposure was through sexual intercourse with an Ebola survivor whose illness had occurred >5 months earlier. His semen was positive by PCR for Ebola virus 199 days after onset of his illness, and Ebola genomic material from both partners shared common mutations (*27*). This patient represented the last Ebola case in the second phase of the Liberia epidemic (*12*). On June 29, 2015, a third epidemic phase began with 6 cases in Margibi and Montserrado Counties without further spread; the origin of infection in the index case-patient was uncertain. Liberia was again declared free of Ebola on September 3, 2015 (*13*).

## Essentials in Containing Ebola, July 2014–September 2015

No single factor explains Liberia’s control of Ebola, and at least 6 issues deserve mention: 1) government leadership and sense of urgency, 2) coordinated international assistance, 3) sound technical work, 4) flexibility guided by epidemiologic data, 5) transparency and communication, and 6) efforts by communities themselves. Instituting the IMS in July 2014 (*14*) was critical for accountability and coordination of multiple partners. The government was always in charge but receptive to external advice channeled through the IMS. The declaration of a state of emergency in August 2014 signaled the gravity of the situation, as did the subsequent closure of land borders with neighboring Sierra Leone and Guinea. Entry and exit screening at airports started in late July 2014, and domestic movement of ill persons was restricted (*28*).

Technical interventions included the early increase in ETU beds in Montserrado County and implementation of burial teams. In Monrovia, isolation of large numbers of patients in late September 2014 and prompt removal of infectious cadavers from the community preceded the documented decrease in cases in the county. Flexibility in response to the changing epidemic was illustrated by the shift in focus from ETUs to CCCs and then to implementation of the RITE strategy, establishment of an IMS for Montserrado County, development of the ring IPC approach, and prioritization of laboratory-confirmed data for guiding interventions.

Community engagement resulted in remarkable behavior change. Physical contact with others ceased; chlorinated handwashing stations sprang up everywhere; and in-country movement reduced. The presidential order for cremation of cadavers in Monrovia was generally respected. By contrast, forcible isolation of case-patients and quarantine of a slum community in Monrovia in August 2014 led to violence, to which the government responded, commendably, with dialogue. Subsequently, voluntary quarantine was instituted only with community agreement and appropriate support, especially provision of adequate food. Many affected communities, some very remote, initiated or supported investigation, contact tracing, and other control efforts with great resilience (*29**,**30*).

Despite selective reporting suggesting discord (*31*), international partners collaborated well with government and the media. The deployment of the US military provided a logistic and psychological boost. Although some individuals and commercial entities left Liberia early and many airlines ceased operations, major organizations including United Nations agencies stayed and Liberia did not feel abandoned. The president and government communicated clearly and honestly. The “Ebola is Real” and “Ebola must Go!” campaigns transmitted critical information and may have contributed to community resistance being less extensive there than elsewhere.

In retrospect, the response would have been enhanced by much greater investment early on in all aspects of data management, including selection of the most appropriate database. Greater efficiency might have been realized with more support for administrative systems such as personnel, payroll, procurement, and logistics though the IMS. The amount of research conducted was limited, reflecting competing demands but also a missed opportunity.

## Staying at Zero and Beyond, May 2015–September 2015

After the Ebola epidemic, the 2 priorities in Liberia are ensuring rapid recognition and containment of resurgent disease and restoring health services to prevent loss of life from traditional concerns such as vaccine-preventable diseases (*32*) or malaria (*33*). The most likely sources of new cases will be importations from Sierra Leone or Guinea, unrecognized transmission chains within Liberia, or sexual transmission from survivors. Reintroduction of the virus from its natural habitat is theoretically possible, and Ebola becoming endemic is a concern.

Border screening and community event–based surveillance in counties bordering Sierra Leone and Guinea have been instituted (*34*). Monrovia is a favored destination for travelers, and tracking of visitors from affected countries has been proposed but may be difficult to implement. Heightened surveillance is indicated in the immediate post-Ebola period. Testing of oral swab samples from cadavers throughout the country should continue until the region is free from Ebola; the value of this practice was demonstrated by recognition of the index case in the third epidemic wave.

Healthcare facilities should maintain clinical suspicion for Ebola and surveillance among healthcare workers, a sentinel population. Simplifying and expanding Ebola testing without all tests triggering ETU admission and contact tracing will be necessary. After approval, Ebola rapid tests could profoundly change clinical practice. Laboratory capacity to distinguish differential diagnoses such as malaria, Lassa fever, yellow fever, and dengue is needed. Enhancement of IPC nationally must continue.

Investment is needed in surveillance, laboratory strengthening, emergency operations center support, epidemiology expertise, outbreak response capacity (including risk communication and health promotion), and the ability to base decisions on data (*35**,**36*). In retrospect, it was lack of such public health systems that enabled the Ebola epidemic to grow in West Africa with such devastating consequences (*36*). A recently evaluated Ebola vaccine may have a role in containing future outbreaks (*37*); priority populations will include high-risk contacts and healthcare workers.

Preliminary observations suggest that about one quarter of Ebola survivors report visual disturbances, the most severe cause being uveitis (*38*), and about half report severe fatigue and joint pains. The medical, psychological, and social sequelae of Ebola should be assessed, including the number and needs of orphans; and medical, psychosocial, and material support provided for survivors. Evidence of sexual transmission (*27*) and prolonged Ebola persistence in semen (*39*) demand study of postrecovery infectiousness for formulation of public health advice.

Ebola survivors in West Africa, who number in the thousands, have suffered stigma and discrimination, now exacerbated by the possibility of sexual transmission. Many Ebola infections resulted from acts of compassion, such as assisting the sick or participating in funerals. Ostracism of survivors would be an unacceptable conclusion to this unique event in global health, the response to which has been a credit to the government and people of Liberia.

## Added in Proof

Since acceptance and publication on-line of this report, a 15-year-old-boy in Montserrado county tested positive for Ebola on November 22, 2015 and died the next day. Two other family members subsequently tested positive and survived.  Rapid response and containment were achieved using the containment strategies and procedures outlined in this report. The source of the cluster was believed to be viral re-emergence in a persistently infected survivor. Liberia was again declared Ebola-free on January 14, 2016.
